# The lack of retropatellar resurfacing at index surgery is significantly associated with failure in patients following patellofemoral inlay arthroplasty: a multi-center study of more than 260 patients

**DOI:** 10.1007/s00167-021-06544-5

**Published:** 2021-04-02

**Authors:** Andreas B. Imhoff, Eva Bartsch, Christoph Becher, Peter Behrens, Gerrit Bode, Matthias Cotic, Theresa Diermeier, Holger Falk, Matthias J. Feucht, Ulrich Haupt, Stefan Hinterwimmer, Johannes Holz, René Hutter, René Kaiser, Tobias Knoblauch, Wolfgang Nebelung, Philipp Niemeyer, Turlough O’Donnel, Geert Pagenstert, Thilo Patzer, Tim Rose, Marco C. Rupp, Thomas Tischer, Arne J. Venjakob, Stephan Vogt, Jonas Pogorzelski

**Affiliations:** 1 Department of Orthopedic Sports Medicine, Klinikum rechts der Isar, Technical University of Munich, Ismaninger Str. 22, 81675 Munich, Germany; 2 ATOS Clinic Heidelberg, Bismarckstr. 9-15, 69115 Heidelberg, Germany; 3 Orthodok, Tonndorfer Hauptstraße 71, 22045 Hamburg, Germany; 4 Gelenkzentrum Rhein-Main, Frankfurter Straße 94, 65239 Hochheim am Main, Germany; 5 Orthomotion, City Clinic Thun, Marktgasse 17, 3600 Thun, Switzerland; 6 OrthoPlus Munich, Alte Börse, Lenbachplatz 2a, 80333 Munich, Germany; 7 Orthocentrum, Hansastr. 1-3, 20149 Hamburg, Germany; 8 Department of Orthopedics, Kantonspital Graubünden, Loëstrasse 99, 7000 Chur, Switzerland; 9 Department of Sport Orthopedics, St. Vinzenz Hospital, Schloßstr. 85, 40477 Düsseldorf, Germany; 10 Department of Orthopedics, University Hospital of Freiburg, Hugstetter Str. 55, 79106 Freiburg, Germany; 11 Center for Orthopaedics, Beacon Hospital, Sandyford, Dublin 18, Dublin, Ireland; 12 Knee Institute Basel, CLARAHOF Clinic of Orthopaedic Surgery, MERIAN-ISELIN-Hospital, Swiss Olympic Medical Center, Föhrenstr. 2, 4054 Basel, Switzerland; 13 Orthopädie-Zentrum, Schön Klinik, Am Heerdter Krankenhaus 2, 40549 Düsseldorf, Germany; 14 Gelenkzentrum Leipzig, Richard-Lehmann-Str. 21, 04275 Leipzig, Germany; 15 Department of Orthopedics, University Hospital of Rostock, Doberanerstr. 142, 18057 Rostock, Germany; 16 Department of Sports Orthopedics, Hessing Stiftung, Hessingstr. 17, 86199 Augsburg, Germany

**Keywords:** Patellofemoral, Knee, Patellofemoral osteoarthritis, Inlay, Patellofemoral arthroplasty, Patellofemoral resurfacing, Trochlea, Retropatellar resurfacing, WAVE prosthesis

## Abstract

**Purpose:**

To evaluate the clinical outcomes of patients with a minimum 2-year follow-up following contemporary patellofemoral inlay arthroplasty (PFIA) and to identify potential risk factors for failure in a multi-center study.

**Methods:**

All patients who underwent implantation of PFIA between 09/2009 and 11/2016 at 11 specialized orthopedic referral centers were enrolled in the study and were evaluated retrospectively at a minimum 2-year follow-up. Clinical outcomes included the Western Ontario and McMaster Universities Osteoarthritis Index (WOMAC) score, the Knee Injury and Osteoarthritis Outcome Score (KOOS), the Tegner Scale, the visual analogue scale (VAS) for pain, and subjective patient satisfaction. Pre- and perioperative risk factors were compared among failures and non-failures to determine potential risk factors.

**Results:**

A total of 263 patients (85% follow-up rate) could be enrolled. The mean age at the time of index surgery was 49 ± 12 years with a mean postoperative follow-up of 45 ± 18 months. The overall failure rate was 11% (28 patients), of which 18% (5 patients) were patients with patella resurfacing at index surgery and 82% (23 patients) were patients without initial patella resurfacing. At final follow-up, 93% of the patients who did not fail were satisfied with the procedure with a mean transformed WOMAC Score of 84.5 ± 14.5 points, a mean KOOS Score of 73.3 ± 17.1 points, a mean Tegner Score of 3.4 ± 1.4 points and a mean VAS pain of 2.4 ± 2.0 points. An increased BMI was significantly correlated with a worse postoperative outcome. Concomitant procedures addressing patellofemoral instability or malalignment, the lack of patellofemoral resurfacing at the index surgery and a high BMI were significantly correlated with failure in our patient cohort.

**Conclusion:**

Patellofemoral inlay arthroplasty shows high patient satisfaction with good functional outcomes at short-term follow-up and thus can be considered a viable treatment option in young patients suffering from isolated patellofemoral arthritis. Patellar resurfacing at index surgery is recommended to decrease the risk of failure.

**Level of evidence:**

Retrospective case series, Level IV.

## Introduction

There is a consensus throughout the literature that the healing capacity of cartilage decreases progressively with advancing age. As a result, total or partial knee arthroplasty is often considered a viable treatment for patients suffering from isolated patellofemoral osteoarthritis when nonoperative treatment modalities, such as physiotherapy, oral analgesics, and activity modification have failed [13]. However, over the past decade, multiple studies have questioned the use of total knee arthroplasty as a primary treatment option for patients with isolated patellofemoral osteoarthritis due to possible overtreatment and thus favored partial arthroplasty [10, 12, 13]. More specifically, isolated patellofemoral arthroplasty using a second-generation inlay trochlear component has become a valid treatment option in recent years [10]. However, as patient numbers were generally small in the published literature due to the rarity of isolated patellofemoral osteoarthritis and as the reported outcomes sometimes contradicted each other, the value of patellofemoral inlay arthroplasty (PFIA) remains unclear [10, 12, 13, 18, 19]. Thus, further well-powered investigations are needed to enhance decision making, enable evidence-based patient counselling and improve clinical practice.

The primary purpose of this retrospective 2-year follow-up multi-center study was to evaluate the clinical results after PFIA. The secondary purpose was to identify potential risk factors which may predispose to failure. The hypothesis was that PFIA results in good and satisfying clinical outcomes, but that the existence of certain risk factors predisposes for failure.

## Methods

### Study population

This was an Institutional-Review-Board (IRB) approved level IV retrospective multi-center study (*each center acquired IRB approval at its respective institution*). A multicenter database was established to evaluate the postoperative outcome with a minimum of 2-year follow-up after PFIA. The data originated from 11 specialized orthopedic referral centers across Europe with long-term experience in the treatment of end-stage patellofemoral osteoarthritis. The study was coordinated by the first author at the *(blinded for review)* and additional data managers were appointed from each center. The data managers of all clinics involved were responsible for collecting and arranging the data in a standardized manner.

A study protocol was designed in consensus with all involved centers and defined the following inclusion and exclusion criteria: all patients suffering from isolated disabling patellofemoral OA (Kellgren-Lawrence grade III–IV [16]) or chondral defects (Outerbridge grade III–IV [24]) which were refractory to conservative treatment and/or failed prior surgical treatment and who consequently underwent PFIA between 09/2009 and 11/2016 using the HemiCAP^®^ Wave Patellofemoral Resurfacing Prosthesis (Arthrosurface, Franklin, MA, USA) with a minimum of 2 years postoperative follow-up were enrolled. Concomitant procedures addressing patellofemoral instability (reconstruction of the medial patellofemoral ligament) or malalignment (high tibial osteotomy or distal femoral osteotomy) were noted for later comparison. Patients were excluded, if they had additional knee surgery unrelated to the patellofemoral joint on the ipsilateral knee, or if they had deceased during follow-up.

### Surgical technique and rehabilitation

All implants were implanted according to the manufacturer`s recommended technique [10]. Circumpatellar denervation and debridement of patellar osteophytes were additionally performed in all cases. Although there were no objective criteria in the decision to resurface the patella across all participating clinics, the majority of the surgeons involved routinely resurfaced the patella. Reasons included severe patellar osteoarthritis and consecutive patellofemoral incongruence caused by focal osteonecrosis or osteolysis with subchondral bone defects and severe patellar dysplasia.

As a part of a structured rehabilitation program, patients were limited to partial weight-bearing of 20 kg for two weeks. Rehabilitation also included manual lymphatic drainage and mobilization was ensured by employing continuous passive motion for the first two weeks. Full range of motion was allowed immediately after surgery. Subsequently, weight-bearing was increased gradually until full weight-bearing was achieved approximately 6 weeks after surgery [10].

### Outcome measurements

Clinical outcomes were evaluated at a minimum of 2-year postoperative follow-up using the Western Ontario and McMaster Universities Osteoarthritis Index (WOMAC)[2], the Knee Injury and Osteoarthritis Outcome Score (KOOS) [26], the visual analogue scale for pain (VAS) on a scale of 0–10, as well as the Tegner Activity Scale. The WOMAC score was subsequently transformed calculating a normalized percentage score (100 indicating no problems and 0 indicating extreme problems) for each subscale. Postoperative patient satisfaction was assessed by a follow-up questionnaire with the options (1) very satisfied, (2) satisfied, (3) partially satisfied, and (4) dissatisfied. Failure of the PFIA was defined as subsequent conversion to total or partial knee arthroplasty during the follow-up period or a transformed WOMAC score less than 43 at final follow up [35].

The association between preoperative characteristics and outcomes including failure was assessed performing a subgroup analysis. The size of our study population statistically limited the number of risk factors to be evaluated, since repeatedly testing an excessive number of factors on a single dataset predisposes for the occurrence of Type 1 (false-positive) errors. Therefore, only the following preoperative factors were selected a priori for assessment of our secondary hypothesis: Constitutional factors (BMI, age, gender), the influence of concomitant procedures, and the influence of primary or secondary patellar resurfacing.

### Statistical analysis

Data analysis was performed using SPSS software version 26.0 (IBM-SPSS, New York, USA). Normally distributed data are reported as mean ± standard deviation, whereas non-normally distributed data are reported as median and range (interquartile range, IQR, from the 25th to the 75th percentile). Spearman’s rank correlation coefficient was used to assess possible correlations between continuous variables and outcome scores. The association between categorical risk factors and failure was assessed using a Chi-squared test while the association between continuous variables and failures was assessed using the Mann–Whitney-*U*-test. The level of significance was set at *p* < 0.05.

## Results

### Study population

**Fig. 1:**
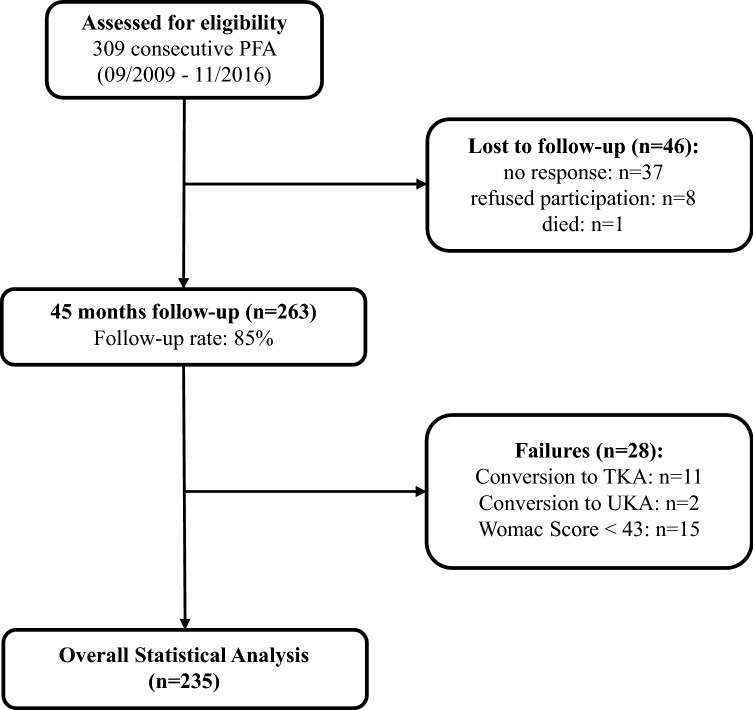
Flow chart of included and excluded patients. *PFA* patellofemoral arthroplasty. *TKA* total knee arthroplasty

Between 09/2009 and 11/2016, a total of 309 patients were treated with PFIA at 11 specialized orthopedic referral centers across Europe. This included 5 centers with more than 20 procedures, one center with 11–20 procedures, two centers with 6–10 procedures, and three centers with 1–5 procedures. A total of 46 patients refused to participate, died during the study period, or could not be reached for follow-up evaluation, leaving 263 patients (85% follow-up rate) enrolled in this retrospective case series. Of those, a total of 28 patients were classified as failures of whom 11 patients had been converted to total knee arthroplasty (TKA), 2 had been converted to unicondylar knee arthroplasty (UKA) and 15 had a transformed overall WOMAC score of less than 43 points (Fig. 1). Revision surgery with secondary resurfacing of the patella was performed in 23 patients (9%) due to persisting anterior knee pain.

### Clinical results

Mean age at the time of surgery was 49 ± 12 years with a mean postoperative follow-up of 45 ± 19 months. The overall failure rate of included patients was 11% (28 patients) of which 18% (5 out of 28 patients) of patients had patella resurfacing at index surgery and 82% (23 out of 28 patients) of patients had not undergone patella resurfacing primarily. Taking into account that 37 patients could not be reached for follow-up and were therefore excluded, the failure rate could potentially be as high as 21.6% (65 out of 300 patients). Patients who failed were included in the risk factor analysis only, as many of them had been converted to TKA before final follow-up. At final follow-up, the vast majority of the patients who did not fail were satisfied with the procedure and reached good functional outcomes at short-term follow-up. The detailed postoperative results at final follow-up of the WOMAC Score, KOOS Score, Tegner Scale, and VAS pain scale as well as detailed characteristics of the patient collective can be found in Table 1.

**Table 1:** Descriptive analysis

Variable	Patient collective
Gender distribution^a^	
Male (*n*, %)	**85 (32%)**
Female (*n*, %)	**178 (68%)**
Age (years)^a^	**49 ± 12**
Body mass index (kg/m^2^)^a^	**26.3 ± 4.6**
Follow Up (months)^a^	**45 ± 19**
WOMAC overall^b^	**81.3 ± 19.0**
WOMAC pain	82.9 ± 20.5
WOMAC stiffness	79.8 ± 23.7
WOMAC function	80.9 ± 19.1
VAS^b^	**2.6 ± 2.3**
KOOS overall^b^	**70.3 ± 20.5**
KOOS pain	79.8 ± 20.3
KOOS symptoms	79.4 ± 18.6
KOOS ADL	80.9 ± 19.0
KOOS SPORT	49.0 ± 27.9
KOOS QDL	62.6 ± 27.2
Tegner^b^	**3.3 ± 1.5**
Subjective satisfaction^b^	
Very satisfied (*n*, %)	117 (47%)
Satisfied (*n*, %)	66 (26%)
Partially satisfied (*n*, %)	45 (18%)
Dissatisfied (*n*, %)	22 (9%)

^a^Entire patient cohort (*n* = 263)

^b^Patient cohort that did not undergo conversion to TKA or UKA (*n* = 250)

No significant difference between centers performing more or less than 10 procedures during the inclusion period could be identified (*p* > 0.05). No significant association between gender or concomitant procedures and postoperative outcome scores could be detected. However, an increased BMI was significantly correlated with worse postoperative outcome scores in the overall KOOS score und Tegner scale. Furthermore, a lower age at surgery was correlated with higher postoperative Tegner activity scores. (Table 2). Furthermore, compared to patients who did not undergo patellar resurfacing at index surgery, patients who underwent patellar resurfacing in the primary procedure and did not fail reported statistically significantly higher transformed overall WOMAC scores (81.9 ± 15.8 vs 86.7 ± 12.8; *p* = 0.011*) and overall KOOS scores (69.5 ± 17.9 vs. 76.7 ± 15.7; *p* = 0.001**).

**Table 2:** Correlation coefficient (Spearman-Rho) between demographic parameters and clinical outcome

	Age (years) (*n* = 235)	Significance	Body mass index (kg/m^2^, *n* = 235)	*p*-value
WOMAC overall	− 0.050	n.s	− 0.127	n.s
VAS	0.008	n.s	0.019	n.s
KOOS overall	0.020	n.s	− 0.164	p = 0.018*
TEGNER	0.143	p = 0.030*	− 0.199	p = 0.004**

*n* number of patients, *kg/m*^*2*^ kilograms per square meter, *n.s.* not significant

** *p* < 0.01; *p < 0.05

### Risk factor analysis for failure

Patient demographics and the presence of risk factors in failures versus non-failures are presented in Table 3. Notably, a significantly higher percentage of patients with concomitant procedures addressing patellofemoral instability or malalignment as well as a higher BMI was identified among the patients who failed compared with those who did not fail. Furthermore, a higher percentage of the patients in the failure group did not undergo patellar resurfacing at index surgery (primary patellar resurfacing). No statistically significant relationship between failures and age or gender could be detected (Table 3).

**Table 3:** Comparison between survivors and failures. Failures were defined as knees who underwent conversion to total knee arthroplasty (TKA) or a Womac Score < 43

Variable	Non-failures (*n* = 235)	Failures (*n* = 28)	*p*-value
Age (years)	49 ± 12	47 ± 10	n.s
Body mass index (kg/m^2^)	26.2 ± 4.6	27.5 ± 3.8	p = 0.045*
Gender distribution			
Male (*n*,%)	78 (33%)	7 (25%)	n.s
Female (*n*,%)	157 (67%)	21 (75%)	
Concomitant procedures			p = 0.025*
No (*n*,%)	205 (87%)	20 (71%)	
Yes (*n*,%)	30 (13%)	8 (29%)	
Patellar resurfacing at index surgery			p = 0.000**
No patellar resurfacing (*n*, %)	111 (47%)	23 (82%)	
Patellar resurfacing (primary) (*n*, %)	124 (53%)	5 (18%)	
Patellar resurfacing			n.s
No patellar resurfacing (*n*, %)	97 (41%)	14 (50%)	
Patellar resurfacing (primary and secondary) (*n*, %)	138 (59%)	14 (50%)	

The patellar resurfacing group was further subdivided between patients who underwent patellar resurfacing at index surgery (primary) and those who underwent implantation of patellar resurfacing as a revision surgery during further follow up (secondary)

Mean values are given with ± standard deviation

*n* number of patients, *kg/m*^*2*^ kilograms per square meter; *n.s.* not significant; *%* percent

**p < 0.01; *p < 0.05

## Discussion

The main finding of this study confirmed our primary hypothesis that PFIA provides satisfying subjective outcomes at short-term follow-up in a selected group of patients. The overall failure rate of 11% within the first 2 years following implantation suggests reliability of the procedure and thus also confirms our primary hypothesis. Patella resurfacing at index surgery further lowered this failure rate to 4%. In general, certain pre- or perioperative characteristics, such as concomitant procedures addressing patellofemoral instability or malalignment, the lack of patellofemoral resurfacing at the index surgery or a high BMI, were predisposing factors for failure in our study, confirming our secondary hypothesis. Moreover, patients presenting with an increased BMI preoperatively and patients not undergoing patellar resurfacing at index surgery were significantly more likely to suffer from a worse postoperative outcome.

The results of this multicenter investigation, observing a favorable postoperative outcome, underscore the previously reported positive effect of the procedure per se in a large patient cohort for the first time [10, 12, 13]. While most of the results following implantation of the HemiCAP^®^ Wave prosthesis range within the outcomes reported across multiple types of patellofemoral arthroplasties in a review of the literature, they surpass the collectively reported data in the transformed WOMAC-scoring [30].

The 2-year failure rate detected in this collective corresponds to the rates reported following implantation of comparable patellofemoral arthroplasty models [6, 30]. This demonstrates the validity of second-generation PFIA as a treatment option for isolated patellofemoral OA with prospects of favorable long-term survival rates. Studies investigating designs of first-generation patellofemoral arthroplasty, for which mid- and long-term follow-up is already available, show survival rates of 84% at a 10-year follow-up [32], 75–80% at 15-year follow-up [17, 30], and 69% at a 20-year follow-up [32]. While the above-mentioned studies provide a possible range for long-term expectations for the HemiCap Wave model, the higher revision rates and lower survival rates of the first generation PFA-designs investigated in these long-term follow-up studies have to be taken into account [8, 25].

Regarding the results of the risk factor assessment, the presence of concomitant procedures addressing patellofemoral instability or malalignment as risk factors for failure are in line with previously published failure analyses. Moreover, previous investigations on patellofemoral malalignment in PFIA found patella alta and patellar subluxation [1] as well as patellofemoral maltracking [36] to be predictive for failure. In the large collective of this study, these findings could be extended to the general necessity for concomitant procedures addressing patellofemoral instability or maltracking. In these cases, concomitant surgery was performed according to an algorithm published by Imhoff et al. [12], to correct anatomical risk factors such as varus/valgus malalignment and insufficiency of the MPFL. The higher failure rate in these cases may root in the biomechanical principle of the patellofemoral inlay prosthesis a priori, as the possibility to intraoperatively correct patellofemoral maltracking is limited. As the medial and lateral trochlear edge are preserved, correction of rotation or alignment in a coronal plane are only possible to a limited extent [1]. Thus, concomitant corrective procedures may fail to fully restore the physiological patellofemoral tracking desirable for optimal biomechanics of the PFIA—especially in cases of complex patellofemoral malalignment [21, 28, 34]. To address this malfunction, an implant design with a larger lateral dimension aimed at enhancing the tracking in complex maltracking pathologies is already available on the market [1].

Similar to our results, an increased BMI was identified as an independent factor predictive for an unfavorable outcome in another PFA model by Liow et al.[20]. As an accepted risk factor for progression in knee OA [9], obesity may predispose for an early conversion to TKA—which remains the main cause for failure in PFA according to the current literature [3, 30, 32].

Not performing concomitant patellar resurfacing at the index surgery was identified as a further significant risk factor for failure. This may follow the rationale that additional patellar resurfacing mitigates the risk of progression of patellar OA and consequently pain—two main reasons for failed PFA treatment [3, 30]. This is supported by the finding, that secondary patellar resurfacing during follow-up of our cohort resulted in an elimination of the risk factor for failure “no patella resurfacing performed” at final evaluation. Indeed, biomechanical studies showed that implantation of a PFA significantly increases contact pressure of the patellofemoral compartment, creating a rationale for additional patella resurfacing [4, 33]. Biomechanical data from Vandenneucker et al. further demonstrated that superior restoration of the physiological kinematics of the patellofemoral joint can be achieved, when patella resurfacing is performed concomitantly [33]. While studies addressing this question in PFA are scarce, extensive review of the literature in TKA demonstrated a lower revision rate when concomitant implantation of a patellar component was performed [11].

With the trend in surgery shifting to treatments of minimal invasiveness, results of modern PFIA treatment nevertheless have been benchmarked against TKA, the established treatment for OA of the knee joint[22]. Biomechanically, PFA can sustain the physiological kinematics of the patellofemoral joint—in contrast to non-physiological conditions in the patellofemoral joint after TKA [27, 33]. Furthermore, it was shown that the ROM [23] and knee extension strength [14] are higher following PFA than TKA. Patient-reported outcomes following PFA were observed to be non-inferior to those reported after TKA while superior results were reported early after surgery [23] and in a young patient collective [15].

With comparable complication rates reported for both procedures in isolated patellofemoral OA [8], PFIA provides advantages over TKA including shorter rehabilitation, less morbidity, shorter intraoperative tourniquet time, preservation of the tibial/femoral bone stock [7, 31] and higher cost-effectiveness in younger patients [5].

While evidence investigating the outcome following patellofemoral arthroplasty has been mounting in recent years, patient satisfaction reporting is still scare [29]. This multi-center study addresses this gap in knowledge the first time in a large patient collective, reporting a high patient satisfaction following PFIA.

While this study does demonstrate interesting findings, it is not without limitations. Firstly, while the data were collected prospectively, the study inherits the associated biases of a retrospective design. No statement about the pre- to postoperative changes could be made as no preoperative clinical scores were available and no control group could be established. Secondly, no radiographic evaluation at the final follow-up was conducted. Thirdly, as surgery was performed by specialists in the treatment of patellofemoral diseases in the respective centers, generalization to treatment with patellofemoral arthroplasty may be limited. Fourthly, there may be a performance bias in surgical technique across 11 different centers. However, benefitting from the comparative aspect of sampling in a multi-center approach may help better reflect general practice and reduce the selection bias of single-center design. Finally, to evaluate the outcome after successful PFIA treatment, failures were excluded from the outcome analysis. This potentially introduces a selection bias but avoids a confounding effect of TKA results. While this study reports outcomes and performs a failure analysis for a short- to mid-term follow-up period, further long-term follow-up is needed to conduct a meaningful comparison to different models of PFA and treatment with TKA.

## Conclusion

Patellofemoral inlay arthroplasty shows high patient satisfaction with good functional outcomes at short-term follow-up and thus can be considered a viable treatment option in young patients suffering from isolated patellofemoral arthritis. Patellofemoral resurfacing at index surgery is recommended for all patients to minimize the risk of failure.

## Abbreviations

BMI: Body mass index
DFO: Distal femoral osteotomy
IRB: Institutional review board
KOOS: Knee Injury and Osteoarthritis Outcome Score
MPFL: Medial patellofemoral ligament
OA: Osteoarthritis
PFA: Patellofemoral arthroplasty
PFIA: Patellofemoral inlay arthroplasty
TKA: Total knee arthroplasty
UKA: Unicompartimental knee arthroplasty
VAS: Visual analogue scale
WOMAC: Western Ontario and McMaster Universities Osteoarthritis Index
